# Positional Changes of the Maxillary Permanent Canines After Treatment with Facemask and Hyrax: A retrospective cohort study

**DOI:** 10.4317/jced.62861

**Published:** 2025-07-01

**Authors:** Jenny Angélica Saldarriaga-Valencia, Adriana Santamaria V, Emery Álvarez-Varela, Luisa Fernanda Villegas-Trujillo, Iván Jiménez, Ruben Darío Manrique-Hernández, Carlos M. Ardila, Ary dos Santos-Pinto

**Affiliations:** 1 Department of Morphology, Genetics, Orthodontics and Pediatric Dentistry, São Paulo State University (UNESP), Araraquara School of Dentistry, Araraquara, São Paulo, Brazil. Pediatric Dentist, CES University, Medellín, Colombia; 2 Postgraduate in Pediatric Dentistry and MSc of Dental Sciences from the CES University, Medellín, Colombia; 3 CES-LPH Research Group, School of Dentistry, CES University, Medellín, Colombia. Professor, Department of Pediatric Dentistry, CES University, Medellín, Colombia; 4 Pharmaceutical Chemistry. MSc & PhD in Epidemiology. CES University, Medellín, Colombia; 5 Department of Periodontics, Saveetha Dental College, Saveetha Institute of Medical and technology sciences, SIMATS, Saveetha. University, Chennai, Tamil Nadu, India; 6 Department of Basic Sciences, Biomedical Stomatology Research Group, Faculty of Dentistry, Universidad de Antioquia U de A, Medellín, Colombia; 7 Department of Morphology, Orthodontics and Pediatric Dentistry, School of Dentistry, São Paulo State University (UNESP), Araraquara, São Paulo, Brazil

## Abstract

**Background:**

Early treatment of Class III malocclusion in growing patients remains a significant orthodontic challenge due to unfavorable growth patterns. This study evaluates the positional changes of the maxillary permanent canines (Mx3) and the first maxillary permanent molars (Mx6) after early treatment of Class III malocclusion with maxillary expansion and protraction (MEP).

**Material and Methods:**

A retrospective cohort study was conducted, including a longitudinal and panoramic radiographic (panorex) analysis. Forty-one Class III patients with maxillary hypoplasia (25 females, 16 males) treated in mixed dentition with MEP were compared to an untreated control group of 41 patients (23 females, 18 males). Positional changes of Mx3 were assessed using panorex. Angulation and sectional location of Mx3 were classified according to Power & Short (PS), the Lindauer modification of Ericson & Kurol (L), and Warford (W).

**Results:**

No statistically significant differences were found in the PS and W analyses or in the height of Mx3 between the initial and final evaluations. The L analysis indicated that most Mx3 were located in sectors I and II. Some Mx3 in sector II progressed toward sector I. No Mx3 in the treated group had a poor prognosis, compared to only 3.7% in the control group. Most Mx3 maintained their L prognosis, with improvement observed in 22% of the control group and 29.3% of the treated group. The majority of Mx3 in the final evaluation had a favorable prognosis.

**Conclusions:**

The results indicate that MEP treatment does not cause unfavorable positional changes in Mx3 or Mx6 in these patients.

** Key words:**Malocclusion, Angle Class III, Palatal Expansion Technique, Extraoral Traction Appliances, Child, Maxillary Permanent Canine, Diagnosis.

## Introduction

Maxillary permanent canines (Mx3) are essential for achieving proper arch shape, symmetry, harmony, and aesthetics. They serve as functional occlusion guides and play a critical role in distributing occlusal and masticatory forces across the maxillary basal bone. Mx3 are the last successor teeth to erupt, with the longest developmental period and most complex eruption path. Due to their tortuous route—from the orbital region to the oral cavity—they frequently exhibit displacements, leading to ectopic eruption or impaction (1). After third molars, Mx3 are the most commonly impacted permanent teeth, with a higher prevalence in females (incidence: 0.8–5.2% in general populations). Bilateral impaction occurs in 17–45% of cases, while palatal impaction accounts for 41–93% of impacted Mx3. Notably, approximately 85% of impacted Mx3 have sufficient space for eruption (1,2).

The etiology of palatal and lingual Mx3 impaction involves both genetic and environmental factors. Genetic predisposition strongly influences impaction risk, but local factors—such as lateral incisor orientation and developmental stage—also determine whether impaction occurs buccally or palatally. Patients with impacted Mx3 typically exhibit a transversely enlarged maxilla but reduced sagittal and vertical dimensions compared to unaffected individuals (1).

Diagnosis of impacted Mx3 relies on clinical assessment (including vestibular protuberance palpation) and imaging, such as panoramic radiography (panorex) and cone-beam computed tomography (CBCT). Panorex delivers 4–15 times less radiation than CBCT, and while CBCT is not recommended as a first-line diagnostic tool, it is indicated when conventional radiography provides insufficient information (3). Systematic reviews report CBCT accuracy at 50–95% versus 39–85% for panorex. However, panorex demonstrates high sensitivity and specificity for oral impactions with Mx3 angulation >65° (4).

The incidence of impacted Mx3 varies by malocclusion class: 9% in Class III, 1.3% in Class II division 1, and 33.5% in Class II division 2 subjects (5). Class III malocclusion prevalence also differs by ethnicity: 3–5% in Caucasians, 14% in Asians (Japanese/Chinese), 5% in Americans, and 9.1% in Latino adolescents (5,6). In Latin America, a study of 4,724 children/adolescents reported a 3.7% prevalence (7).

Early treatment of Class III malocclusion in growing patients remains a significant orthodontic challenge due to unfavorable growth patterns. Growth modification therapies—such as maxillary protraction, functional regulators, and chin cups—are most effective when initiated in mixed dentition before the pubertal growth peak (8). Combined maxillary expansion and protraction (MEP) is the gold standard, producing skeletal improvements but also undesired dentoalveolar effects (e.g., maxillary incisor proclination, anterior displacement/extrusion of first molars-Mx6) (9).

Maxillary hypoplasia is present in 25% of Class III cases, with another 25% exhibiting combined maxillary hypoplasia and normal/mild mandibular prognathism. Untreated Class III malocclusion worsens due to adverse skeletal growth and compensatory dental changes (10). Early intervention is crucial to achieve balanced sagittal/transverse relationships and reduce the need for complex orthodontics or orthognathic surgery (11).

Panorex is the primary diagnostic tool for patients aged 8-10 years, enabling evaluation of Mx3 positional changes. At this stage, Mx3 migrates along the distal surface of the maxillary lateral incisor (Mx2). Panorex aids in eruption monitoring, impaction prediction, and assessment of Mx3 inclination (vertical/horizontal) and proximity to the midline, which are critical for diagnosing impaction risks and planning interventions (12).

This study evaluates the positional changes of Mx3 and Mx6 following early Class III treatment with maxillary expansion and protraction (MEP).

## Material and Methods

- Participants

This study has a retrospective cohort design with a longitudinal analysis. The Institutional Human Research Ethics Committee at CES University, Medellín, Colombia approved the protocol.

This investigation was performed with patients diagnosed with skeletal class III due to maxillary hypoplasia, who were treated with MEP. They were submitted to the same treatment protocol, a Hyrax type of expander and Petit type facial mask for MEP, in a private practice of three standarized pediatric dentists.

A control group of growing patients without treatment, similar in age, sex, ethnical background and dentition to the treated group was used to discriminate the changes caused by treatment from those resulting from the normal pattern of facial growth and dental development.

Treated Group: Patients whose cephalic and clinical presented a class III skeletal relationship due to maxillary hypoplasia, anterior crossbite or edge-to-edge incisal relationship, ANB angle less than 3°, Witts of 1.5mm or less, without dental extractions, mixed dentition, skeletal maturation CS1 and CS2 prepubertal and without previous maxillary orthopedic treatments.

Exclusion criteria: patients who presented syndromes, maxillary dental anomalies, early maxillary dental losses, craniofacial anomalies, the presence of class III malocclusion due to mandibular prognathism or no parental consent to participate.

Sample characteristics of the group treated: Radiographic exams of 41 patients who underwent a treatment protocol of their class III skeletal relationship due to maxillary hypoplasia similar to that described by Turley (13).

**Figure 1:**
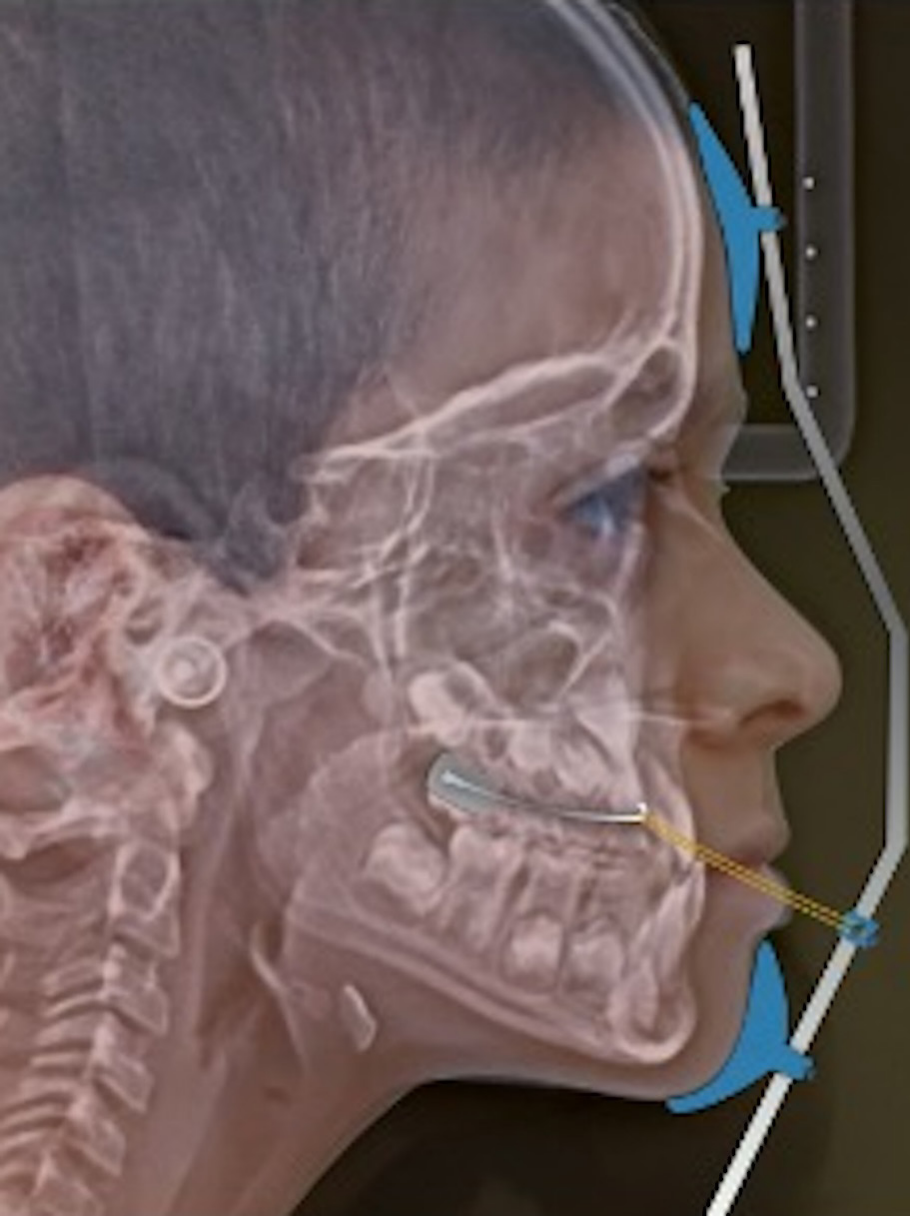
Hyrax type screw fixed on bands in the first maxillary permanent molars (Mx6) and with vestibular hooks at the canine level for face mask protraction (FMP).

The treatment protocol consists in rapid palatal expansion (RPE) performed using a Hyrax type screw fixed on bands in the Mx6 and with vestibular hooks at the canine level for face mask protraction (FMP) (Fig. 1). Hyrax screw was activated a quarter turn by day for 20 days until reaching the appropriate transverse relationship according to the initial condition of each patient. A Petit-type face mask was used for maxillary protraction for 14 to 16 hours per day with elastics that generated 16 ounces of force (300 to 500 grams) per side and with an anterior and inferior force vector 30 to 40 degrees with respect to the occlusal plane. The face mask was used actively until correcting the anterior crossbite and facial aesthetics of the patient.

Data were derived from the evaluation performed at the start and at the end of treatment.

Control Group: Derived from a radiographic original sample of 41 subjects with normal facial features and no history of orthodontic treatment from the CES University Craniofacial growth sample, with an 18-year follow-up (14).

The 41 panorex selected for the control group were comparable in age, gender ( Table 1), and dentition phase ( Table 1), to the treated group before and after treatment, to assess the natural positional changes of Mx3 resulting from normal dentofacial growth. The mean evaluation time was 1.6 ± 0.7 years (1.6 ± 0.6 years for the control group and 1.6 ± 0.8 years for the treated group).

Analysis Pre calibration: before data collection, a calibration process was conducted between the researcher and an expert professional considered as the gold standard and using a set of measurements of 15 subjects, six comparisons were made. An overall Cohen Kappa index of 89% among the observers were obtained indicating an excellent degree of agreement and the researcher was ready to start the data collection.

Error and bias control: All radiographs were traced and measured by the same operator after the pre calibration process. Intra-operator error was evaluated by repeated measurement in 10% of the radiographs chosen randomly. Cronbach’s alpha statistics obtained were between 0.890 and 0.998, and paired t-tests for all variables were less than 0.5 with not significant differences (*p*< 0.05), indicating excellent reliability with acceptable random and systematic errors.

**Figure 2:**
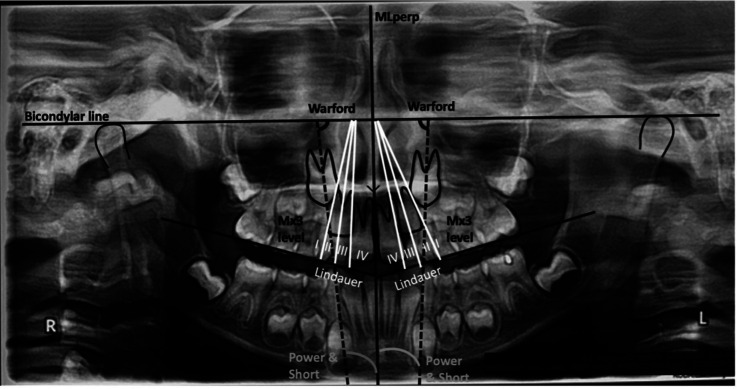
The position of the Mx3. The level, angulation, and sectional location of the Mx3 according to authors Power & Short (PS), Lindauer *et al*. (L) and Warford *et al*. (W) criteria.

Variables: The angulation and sectional location of the Mx3 classified the positional changes of them according to authors Power & Short (PS) (15), Lindauer *et al*. (L) (16) modification of Ericson & Kurol (17) and Warford *et al*. (18) (W) criteria (Fig. 2). These measurements were obtained to determine whether the position of the Mx3 had a favorable, fair, or poor eruption prognosis. In addition, the measurement of the height of the Mx3 in relation to the occlusal plane (Mx3 Level) and its degree of root development and angulation of the Mx6 and its degree of root development were obtained from panorex.

Analysis of Power & Short (PS): Measure the angulation of the canine by the angle formed between the longitudinal axis of the canine to the referential midline perpendicular (MLperp = perpendicular to the edge of the radiograph that passes through the anterior nasal spine). When the angle is between 0 and 15 degrees the prognosis is favorable; between 15 and 30 degrees is regular; and when it exceeds 31 degrees, the prognosis is poor.

Analysis of Lindauer *et al*. (16), modification of the Ericson & Kurol analysis (L) (17): Classify the position of the Mx3 into 4 sectors, defined by three tangent lines using the lateral incisor as reference (tangent lines to its mesial, distal surface and central bisector).

Sector I: located distal to the distal tangent.

Sector II: located between the distal and bisector tangents.

Sector III: located between the mesial and bisector tangents.

Sector IV: located mesial to the mesial tangent.

When the canine is in sectors III and IV, it is considered with the incidence of impaction. The prognosis is favorable when the cusp tip of the canine is in sector I and worsens the more mesial its cusp is located.

Analysis of Warford *et al*. (W) (18): establishes the prognosis of Mx3 eruption based on the angulation formed between a bicondylar line drawn on the panoramic radiograph and the longitudinal axis of the Mx3.

The prognosis is favorable when the angle is greater than 75°; regular when it is between 75° and 59°; and poor when it is less than 59° Mx3 Level: measures the distance (height) from the vertex of the canine to the occlusal plane. The level of Mx3 eruption is obtained from its height in relation to the incisal edges (occlusal plane). The higher it is, the more likely it is to be impacted.

**Figure 3:**
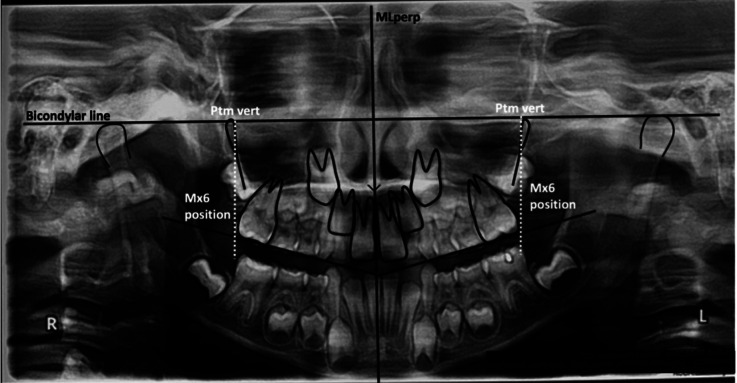
Mx6 position (maxillary first permanent molars position).

Mx6 (maxillary first permanent molars) position: The distance of the Mx6 with respect to the Pterygomaxillary (ptm) fossa was established by the distance in millimeters of the most posterior (distal) point of the Mx6 to the vertical line ptm fossa (vertical line that passes through the most inferior point of the ptm fossa) perpendicular to the bicondylar line. This distance represents the changes in the sagittal position of the Mx6 after treatment with Hyrax and facial mask in the treated group and the sagittal position of the Mx6 in the control group according to the natural growth pattern in the untreated individual (Fig. 3).

Mx6 and Mx3 degree of root development according to Nolla: The stage of root development according to Nolla is also determined for the Mx6 and Mx3 in the patients with mixed dentition. The onset of dental eruption coincides with stage 7 of root formation and the emergence of tooth in the mouth when three-quarters of the root have been formed between stages 8 and 9.

Statistical analysis: The data were analyzed using paired t-test to assess the differences in the variables before and after the treatment (for dependent samples) and between the treated and control groups (Levene test was used to verify the homogeneity of the variances and the corresponding t-test for independent samples). The chi-square test was used to analyze the association between categorical variables intra and inter-groups. All comparisons for hypothesis testing were made with a significance of 0.05.

Power analysis: Sample size analysis resulted in 87.7% of power performed for a group of 41 individuals with an alpha of 0.05 and effect size of 0.5 for the students t-test for dependent samples and 60.9% of power for two groups of 41 individuals with an alpha of 0.05 and effect size of 0.5 for the paired t-test for independent sample. For the Pearson chi-square for a group of 41 individuals with an alpha of 0.05 and effect size of 0.5 resulted in a power of 77.2% between initial and final evaluation and 82 individuals with an alpha of 0.05 and effect size of 0.5 resulted in a power of 97.8% between control and treated groups) (G*power version 3.1.9.4 University of Kiel, Germany).

## Results

Angulation Analysis (PS and W)

Both Power & Short (PS) and Warford (W) analyses demonstrated consistent trends in maxillary canine (Mx3) angulation ( Table 2). The Mx3 became more horizontally aligned over time, evidenced by a significant decrease in angulation (PS: increased angle; W: decreased angle) in the control group (*p* < 0.05), though this change was non-significant in the treated group. Despite the treated group exhibiting more horizontal Mx3 overall, both groups maintained a favorable eruption prognosis (W > 75°; PS: 0–15°). The vertical position (Mx3 Level) decreased significantly with growth (control: 4.2 mm; treated: 3.2 mm), with no intergroup differences (*p* > 0.05).

First Molar (Mx6) Displacement

At baseline, the control group exhibited more anteriorly positioned and less distally inclined Mx6 compared to the treated group. Both groups showed similar anterior displacement (2.2 mm), preserving the initial intergroup difference (2.6 mm). Treatment resulted in a marginally greater reduction in distal inclination (treated: 1.9°; control: 1.5°).

Sector Analysis (Lindauer, L)

Most Mx3 were located in Sectors I and II (favorable/regular prognosis) in both groups ( Table 1). By the final evaluation, some Mx3 in Sector II progressed to Sector I, improving their prognosis. No treated-group Mx3 were in Sectors III/IV (high impaction risk), while 3.7% of control-group Mx3 occupied these sectors.

Prognosis Distribution (PS, L, W)

PS Analysis ( Table 1):

Control group: Favorable prognosis decreased from 81.7% to 68.3%, with a rise in regular prognosis (15.8% → 30.5%). Unfavorable cases were rare (initial: 2 canines; final: 1).

Treated group: Minimal change in favorable prognosis (73.2% → 71.9%); regular prognosis increased slightly (25.6% → 28%). Only one unfavorable case resolved by final evaluation.

L Analysis ( Table 1):

Both groups showed improved favorable prognosis (control: 54.9% → 74.4%; treated: 52.4% → 75.6%) and reduced regular prognosis. Unfavorable cases persisted only in the control group (3 canines).

W Analysis ( Table 1):

Control group: Favorable prognosis declined (78.0% → 63.4%); regular prognosis increased (19.5% → 35.4%).

Treated group: Favorable prognosis improved (67.1% → 70.7%).

Final Prognosis Comparison

Post-treatment/observation, both groups exhibited comparable eruption prognoses ( Table 3):

Favorable: 63.4–75.6%

Regular: 21.9–35.4%

Unfavorable: Only in controls (1.2–3.7%).

Prognosis Evolution

Most Mx3 maintained their initial prognosis (65.5–73.2% across analyses;  Table 4).

Key trends:

Improvement: More frequent in the treated group (L analysis: 29.3% vs. 22% in controls).

Worsening: Higher in controls (PS/W: 22–24.4%) than treated (13.4–15.9%).

Root Development (Nolla’s Stages)

Mx3: Progressed from stages 6–8 (initial) to 7–9 (final) in both groups.

Mx6: Advanced from stages 7–9 to 8–10 (T Table 1).

## Discussion

Early diagnosis of altered maxillary permanent canine (Mx3) eruption is crucial for pediatric dentists and orthodontists to implement appropriate management strategies for potential impaction. Impacted Mx3 requires complex and prolonged treatment due to its challenging nature (19). While this condition may occur with any malocclusion type, it appears in 9% of Class III malocclusion cases, which affects 3.7-9.1% of Americans and 14% of Asians (5-7).

Diagnosis of Mx3 impaction can be established by age 8 using geometric measurements on panoramic radiographs (panorex) (20). Panorex provides essential diagnostic information for monitoring eruption patterns and predicting impaction likelihood. This imaging modality enables evaluation of the Mx3’s vertical position relative to the occlusal plane, its angulation in both vertical and horizontal dimensions, and its proximity to the midline - all critical for assessing eruption trajectory (4,12,20).

The study sample comprised ethnically matched participants: children with Class III malocclusion due to maxillary hypoplasia in the treatment group, and controls with normal facial features matched by gender and age from a craniofacial growth study (14). All participants were in CS1 or CS2 cervical vertebral maturation stages and the mixed dentition phase, primarily the early mixed dentition period - representing an optimal developmental stage for initiating treatment (21).

Maxillary protraction with facemask therapy represents the most frequently employed treatment for Class III malocclusion correction (9,21). While effective for skeletal modification, this approach induces certain dentoalveolar effects including maxillary incisor proclination, anterior displacement and extrusion of maxillary molars, as well as lingual inclination of mandibular incisors. Clinical experience demonstrates that patients undergoing facemask treatment for Class III malocclusion with maxillary hypoplasia frequently require subsequent interventions to properly position the maxillary permanent canine (Mx3) due to space deficiencies (22,23). These observations underscore the importance of evaluating Mx3 position prior to initiating protraction therapy, regardless of apparent eruption abnormalities, as emphasized by Ferreira *et al*. (19).

Angular measurements using Power & Short (PS) and Warford (W) analyses assessed Mx3 orientation relative to the midline perpendicular and bicondylar line, demonstrating consistent trends. Both methods revealed decreasing Mx3 angulation over time, with significant horizontalization in controls but stable measurements in treated cases ( Table 2). Although treated Class III subjects exhibited more horizontally positioned Mx3 than Class I controls at baseline, both groups maintained predominantly favorable eruption prognoses. These findings align with Sajnani and King’s work (2) documenting the Mx3’s natural transition from mesial to vertical alignment between 8-9 years of age, guided by adjacent lateral incisor root development. Our cohort (ages 6-8 years) displayed this characteristic shift from increasing mesial angulation at age 8 toward vertical orientation and midline parallelism by age 9 (2). The authors highlight genetic factors and lateral incisor root guidance (or lack thereof) as critical determinants of buccal versus palatal impaction (1,2).

Eruption predictability varies according to angular characteristics, as Ericson & Kurol (17) and Power & Short (15) reported reduced eruption likelihood with more perpendicular Mx3 angulation relative to the midline. Sajnani & King (2) identified vertical cusp tip position relative to the occlusal plane as the most reliable predictor. However, several authors caution that linear measurements and angular assessments demonstrate limited value for predicting interceptive treatment outcomes, treatment duration, or periodontal status (9,16).

Vertical position analysis revealed no intergroup differences in Mx3 level. During the 1.6±0.7-year observation period, mean Mx3 cusp tip-to-occlusal plane distance decreased by 4.16mm (treated) and 3.19mm (controls). While 73-80.5% of cases showed favorable positional improvement, 5% exhibited increased measurements and 12.2-22% demonstrated no change - both considered unfavorable patterns. These results corroborate Di Carlo *et al*. (24) and Mercuri *et al*. (25), who found no significant differences in Mx3 impaction prevalence or association with Class III skeletal characteristics.

Spatial relationships significantly influence impaction outcomes, as Uribe *et al*. (12) demonstrated that complete Mx3 development, midline angulation, and lateral incisor overlap serve as reliable impaction indicators. Sector location and angulation predict eruption success following deciduous canine extraction, with mesial overlap degree strongly impacting impaction severity (12,18).

Lindauer’s analysis of our data revealed that the majority of maxillary permanent canines (Mx3) were positioned in Sector I (good prognosis) and Sector II (fair prognosis) in both study groups. During final evaluation, some Mx3 originally in Sector II progressed to Sector I, demonstrating improved positioning. Notably, no Mx3 in the treated group were found in Sectors III or IV (poor prognosis), while the control group showed minimal representation (3.7%) in these unfavorable sectors ( Table 1).

Prognosis stability was observed in most cases, with 73.2% of control group Mx3 and 64.6% of treated group Mx3 maintaining their initial classification. Improvement occurred in 22% of control and 29.3% of treated cases, while deterioration was minimal (4.9% control, 6.1% treated) ( Table 3).  Table 1 confirms that the majority of Mx3 ultimately achieved favorable prognosis regardless of treatment status.

In growing Class III patients, Mx3 eruption disturbances frequently occur due to maxillary arch space deficiencies. While some clinicians associate these impactions with maxillary protraction therapy (22), other studies including ours demonstrate no significant positional changes attributable to facemask treatment (26). Our findings align with Tepedino *et al*. (23), confirming that maxillary protraction does not increase Mx3 impaction risk in mixed dentition patients with maxillary hypoplasia.

Maxillary expansion induces forward and downward maxillary displacement through effects on intermaxillary and circummaxillary sutures, enhancing the skeletal response to protraction forces (27). The therapeutic mechanism involves directed force application to weakly fused circummaxillary sutures in young patients, stimulating sutural bone apposition (26). This approach simultaneously addresses posterior crossbite and maxillary disarticulation. Both facemask therapy alone and combined with rapid maxillary expansion (RME) effectively improve Class III malocclusions, suggesting RME application should follow clinical indications rather than serve solely as an adjunct to Class III correction (26). Baccetti *et al*. (28) proposed RME as an interceptive treatment for Mx3 impaction, demonstrating 65.7% eruption success versus 13.6% in controls (*p*<0.001), potentially through intraosseous canine position improvement (9). Ethnic variations exist, with Chinese populations showing buccal impactions associated with anterior transverse deficiencies and palatal impactions linked to lateral incisor anomalies (27).

Maxillary first molar (Mx6) evaluation revealed greater distal positioning and inclination in treated patients at baseline. Both groups showed comparable anterior displacement (2.2 mm), maintaining the initial 2.6 mm intergroup difference. Treatment slightly reduced distal inclination (1.9° vs 1.5° in controls), suggesting Hyrax anchorage minimized molar tipping and preserved anterior space for canine alignment ( Table 1). Interestingly, Mx3 angulation remained lower in controls throughout observation ( Table 1).

Root development analysis challenges the hypothesis linking delayed dental maturation to Mx3 impaction. While Sigler *et al*. (29) reported reduced eruption success with complete root formation (Nolla stage 9-10), Uribe *et al*. (12) emphasize dental age multifactoriality, and Lovgren *et al*. (30) found no clear association between delayed development and palatal impaction. Our study showed similar developmental progression between groups, with molars consistently more advanced than canines. Complete Mx3 root formation was uncommon (control:17.1%; treated:4.9%) at final evaluation ( Table 1).

Our findings demonstrate that maxillary protraction therapy does not induce unfavorable positional changes in either maxillary permanent canines (Mx3) or first molars (Mx6) among mixed dentition patients with Class III malocclusion due to maxillary hypoplasia. These results align with the conclusions of Tepedino *et al*. (23), who reported no increased risk of Mx3 impaction following maxillary protraction across three treatment modalities (Rapid Palatal Expansion alone, RPE with facemask, or Class III functional appliances) in patients at CS1-CS3 skeletal maturation stages. Both our data and existing literature suggest that favorable pretreatment characteristics - including less severe canine displacement sectors (I-II), prepubertal skeletal maturity (CS1-CS2), and open root apices - serve as reliable predictors for successful canine eruption.

This study has several limitations inherent to its retrospective design and reliance on panoramic radiography (panorex) for evaluation. Furthermore, the 1.6-year follow-up period precludes definitive conclusions regarding final eruption outcomes or impaction occurrence in our sample population.

## Conclusions

The current study demonstrates that maxillary expansion and protraction treatment does not adversely affect maxillary permanent canine or first molar positions in mixed dentition patients with Class III malocclusion due to maxillary hypoplasia. Both angulation analyses (Power & Short and Warford) showed comparable favorable prognoses between treated and control groups, while Lindauer sector analysis revealed most canines maintained or improved their eruption potential. While treated cases exhibited initially more distally positioned first molars, both groups demonstrated similar anterior displacement patterns with treatment. These findings support the safety of early facemask therapy for Class III correction without increasing impaction risks, though longer-term evaluation would strengthen these conclusions.

## Figures and Tables

**Table 1:** Demographic, dental, and canine eruption analysis (Lindauer, Power & Short, Warford) with Nolla stages for Mx3 and Mx6 in control and treated groups.

Variable	Category	Control (N=41)	Treated (N=41)	p-value (if applicable)
Age (years)	Female (Mean ± SD)	7.1 ± 1.3	7.0 ± 1.3	NS
	Male (Mean ± SD)	7.1 ± 1.1	7.4 ± 1.3	NS
Dentition	Mixed 1st period (%)	82.9	85.3	NS
	Mixed intermediate (%)	2.5	12.2	
	Mixed 2nd period (%)	14.6	2.5	
Lindauer (Sector)	Sector I final (%)	74.4	76.8	0.025 (Ctrl) / 0.002 (Tr)
	Sector II final (%)	22.0	23.2	
Power & Short	Favorable final (%)	68.3	71.9	<0.001 (Ctrl) / 0.061 (Tr)
Warford	Favorable final (%)	63.4	70.7	<0.001 (Ctrl) / 0.017 (Tr)
Nolla Mx3 (Final)	Stage 7-9 (%)	90.2	98.8	
Nolla Mx6 (Final)	Stage 9-10 (%)	61.0	80.5	

**Table 2:** Descriptive Statistics: Power & Short (PS), Warford analysis (W), the level of the maxillary permanent canines (Mx3), angulation and position of the maxillary first permanent molars (Mx6) in the initial e final evaluation according to the group.

Group								Control x Treated	
		Control			Treated			Difference	
Analysis	Evaluation	n	Mean	SD	n	Mean	SD	Mean	SE
Power & Short (PS)	Initial	82	9.97	7.12	82	11.33	7.24	1.36ns	1.12
	Final	82	12.68	8.70	82	12.70	6.60	0.02ns	1.21
	Differencea		2.71*	1.04b		1.37ns	0.95b		
Warford (W)	Initial	82	79.86	7.23	82	78.65	7.75	-1.21ns	1.17
	Final	82	77.24	9.12	82	77.43	7.28	0.18ns	1.29
	Differencea		-2.62*	1.08b		-1.22ns	1.03b		
Mx3 Level	Initial	82	17.85	3.14	82	17.29	2.69	-0.56ns	0.46
	Final	82	13.69	6.69	82	14.10	4.37	0.41ns	0.88
	Differencea		-4.16**	0.57b		-3.19**	0.34b		
Mx6 Angulation	Initial	82	102.26	8.80	82	105.32	7.08	3.05*	1.25
	Final	82	100.77	6.69	82	103.40	6.04	2.63**	1.00
	Differencea		-1.49ns	1.13b		-1.91**	0.72b		
Mx6 Position	Initial	82	8.38	3.20	82	5.76	3.80	-2.62**	0.55
	Final	82	10.67	3.97	82	8.02	3.86	-2.64**	0.61
	Differencea		2.28**	0.47b		2.26**	0.41b		

a Difference between Initial and final evaluation; b SE; * *p* < 0.05; ** *p* <0.01; ns not significant p> 0.05.

**Table 3:** Pearson chi-square test for group comparison (Control x Treated) of the prognosis of Mx3 eruption according to PS, L and W analysis in the final evaluation. Frequency (percentage).

Power & Short (PS) Prognosisa	Unfavorable	Regular	Favorable	Total
Control	1(1.2)	25(30.5)	56(68.3)	82(100)
Treated	0	23(28.0)	59(72.0)	82(100)
a Chi-square 4.758 (Sig 0.093);				
Lindauer (L)Prognosis b	Unfavorable	Regular	Favorable	Total
Control	3(3,7)	18(21.9)	61(74.4)	82(100)
Treated	0	20(24.4)	62(75.6)	82(100)
b Chi-square 3.555(Sig 0.169)				
Warford (W) Prognosis c	Unfavorable	Regular	Favorable	Total
Control	1(1.2)	29(35.4)	52(63.4)	82(100)
Treated	0	24(29.3)	58(70.7)	82(100)
c Chi-square 1.923 (Sig 0.382)				

**Table 4:** Evolution prognosis of Mx3 eruption according to PS, L and W in the control and treated groups. Frequency (percentage).

Power & Short (PS) Prognosisa	Worsened	Unchanged	Improve	Total
Control	18(22.0)	57(69.5)	7(8.5)	82(100)
Treated	13(15.9)	57(69.5)	12(14.6)	82(100)
a Chi-square 2.122 (Sig 0.436)				
Lindauer (L) Prognosis b	Worsened	Unchanged	Improve	Total
Control	4(4.9)	60(73.2)	18(22.0)	82(100)
Treated	5(6.1)	53(64.6)	24(29.3)	82(100)
b Chi-square 1.402(Sig 0.496)				
Warford (W) Prognosis c	Worsened	Unchanged	Improve	Total
Control	20(24.4)	54(65.9)	8(9.8)	82(100)
Treated	11(13.4)	57(69.5)	14(17.1)	82(100)
c Chi-square 4.330 (Sig 0.115)				

## Data Availability

The datasets used and/or analyzed during the current study are available from the corresponding author.
